# Late-Diagnosed Cystic Fibrosis in an Octogenarian With an Atypical Phenotype of Pancreatic Sufficiency and Preserved Pulmonary Function

**DOI:** 10.7759/cureus.114296

**Published:** 2026-08-10

**Authors:** Brenda Hranec, Luke Hudson, Daniel Layish

**Affiliations:** 1 College of Medicine, Florida State University, Tallahassee, USA; 2 Pulmonary Medicine, AdventHealth Orlando, Orlando, USA

**Keywords:** bronchiectasis, cftr modulator, chronic sinusitis, cystic fibrosis, elderly, late diagnosis, octogenarian, pancreatic sufficiency, pseudomonas aeruginosa

## Abstract

Cystic fibrosis (CF) was historically described as a severe childhood disease associated with pancreatic destruction, malnutrition, recurrent respiratory infection, and early mortality. Advances in sweat chloride testing, cystic fibrosis transmembrane conductance regulator (CFTR) gene discovery, newborn screening, multidisciplinary care, and CFTR modulator therapy have transformed the natural history of the disease. However, CF remains clinically heterogeneous, and patients with residual CFTR function may present later in life with chronic bronchiectasis, sinus disease, atypical respiratory infections, or nonspecific gastrointestinal symptoms. Diagnosis in advanced age is uncommon and may be delayed when pancreatic function is preserved and sweat chloride values are intermediate.

An 87-year-old woman with late-diagnosed cystic fibrosis was followed longitudinally for chronic sinopulmonary disease. She had a history of recurrent sinus infections and was evaluated at a tertiary institution, where CF was diagnosed at approximately age 62. Her family history was notable for a maternal aunt who died from an unspecified respiratory illness. Diagnostic evaluation demonstrated an intermediate sweat chloride value of 41 mmol/L and CFTR testing showing one copy of F508del, M470V polymorphism, and a rare C76W variant. Her phenotype included chronic bronchiectasis, chronic sinusitis with nasal polyps, two prior sinus surgeries, and prior *Mycobacterium avium *complex infection treated in 1995. She had pancreatic sufficiency, with normal fecal elastase and no clinical response to pancreatic enzyme replacement therapy. Pulmonary function testing showed preserved lung function with stage I obstruction, including FEV1 1.33 L, 89% predicted; FVC 1.95 L, 85% predicted; and FEV1/FVC ratio of 68%, stable compared with the prior year. Her course was also notable for osteoporosis with prior vertebral compression fractures, which limited tolerance of vest-based airway clearance. Management included airway clearance with an oscillatory positive expiratory pressure device, individualized bronchodilator therapy due to tachycardia with albuterol, surveillance respiratory cultures, nutritional and weight monitoring, otolaryngology follow-up for chronic sinusitis and nasal polyps, osteoporosis management, and treatment with vanzacaftor/tezacaftor/deutivacaftor, to which she reported symptomatic improvement. This case highlights an uncommon late-diagnosed CF phenotype in an octogenarian with pancreatic sufficiency, intermediate sweat chloride, chronic sinopulmonary disease, preserved lung function, and new isolation of *Pseudomonas aeruginosa*. The case emphasizes that preserved pancreatic function and advanced age do not exclude CF and that adult patients with chronic bronchiectasis, sinus disease, and atypical respiratory infections should be evaluated for CFTR dysfunction when clinically appropriate.

## Introduction

Cystic fibrosis (CF) is an autosomal recessive disorder caused by pathogenic variants in the cystic fibrosis transmembrane conductance regulator (CFTR) gene, resulting in impaired chloride and bicarbonate transport across epithelial surfaces [1,2]. Although CF was historically described as a severe childhood disease associated with pancreatic involvement, malnutrition, chronic respiratory infection, and early mortality [3], the clinical spectrum is now recognized to be substantially broader. CF affects an estimated 34,526 individuals receiving care at accredited centers in the United States [4].

Individuals with residual CFTR function may present later in life with atypical or milder phenotypes, including chronic bronchiectasis, recurrent sinus disease, nasal polyps, nontuberculous mycobacterial (NTM) infection, pancreatitis, or nonspecific gastrointestinal symptoms [5,6]. These patients are more likely to retain pancreatic sufficiency and may have intermediate sweat chloride values, making diagnosis more dependent on careful clinical correlation and CFTR genetic testing [2].

Historically, management has consisted of therapy targeted at symptom management applied on the basis of clinical presentation rather than underlying genotype. In infants and young children, care emphasizes early nutritional assessment, pancreatic function testing, pancreatic enzyme replacement therapy, supplementation of fat-soluble vitamins and salt, growth monitoring, infection prevention, and initiation of age-appropriate airway clearance [6]. As patients grow older, treatment may expand to include regular airway-clearance techniques, inhaled hypertonic saline or dornase alfa to improve mucus clearance, bronchodilators when clinically indicated, and culture-directed oral, inhaled, or intravenous antibiotics for airway infection [6]. Chronic inhaled anti-pseudomonal antibiotics and azithromycin may be considered in selected patients, while advanced disease may require supplemental oxygen, noninvasive ventilation, management of hemoptysis or pneumothorax, and lung-transplant evaluation [6].

Recognition of adult-diagnosed CF is increasingly important because CF-specific surveillance, airway clearance, infection monitoring, nutritional support, bone health management, and genotype-directed CFTR modulator therapy may still provide clinical benefit. Highly effective CFTR modulators have changed the disease course for eligible patients, and newer regimens such as vanzacaftor/tezacaftor/deutivacaftor expand once-daily modulator options for individuals with responsive CFTR variants [7-9].

We present the case of an 87-year-old woman diagnosed with CF at approximately age 62 after evaluation at a tertiary institution for recurrent sinus infections and chronic sinopulmonary disease. Her course is notable for pancreatic sufficiency, chronic sinusitis with nasal polyps, bronchiectasis, prior *Mycobacterium avium* complex (MAC) infection, osteoporosis, preserved lung function with stage I obstruction, and clinical response to vanzacaftor/tezacaftor/deutivacaftor. This case highlights that CF should remain in the differential diagnosis for adults with longstanding sinopulmonary disease, even in the absence of pancreatic insufficiency and even at advanced age.

## Case presentation

An 87-year-old woman with late-diagnosed CF was followed longitudinally for chronic sinopulmonary disease. She had experienced recurrent sinus infections for many years and was ultimately evaluated at a tertiary institution, where CF was diagnosed at approximately age 62. Her family history was notable for a maternal aunt who died from an unspecified respiratory illness, although there was no known confirmed family history of CF. 

Diagnostic evaluation demonstrated an intermediate sweat chloride value of 41 mmol/L. CFTR testing showed one copy of F508del, M470V polymorphism, and a rare C76W variant. Her clinical phenotype included chronic bronchiectasis, chronic sinusitis with nasal polyps, and a history of two prior sinus surgeries. She also had a prior MAC infection treated in 1995, as well as previous airway growth of methicillin-sensitive *Staphylococcus aureus* and *Stenotrophomonas maltophilia*.

Although her clinical features were compatible with CF, the available data did not meet Cystic Fibrosis Foundation consensus criteria [2], which require a sweat chloride concentration of 60 mmol/L or greater, two CF-causing variants in trans, or abnormal nasal potential difference or intestinal current measurement. Accordingly, the patient is described throughout this report as having CF on the basis of her clinical diagnosis that was established at an outside institution. 

The patient was pancreatic sufficient. Prior fecal elastase testing was normal at 241 µg/g, and she did not experience clinical improvement with pancreatic enzyme replacement therapy. This pancreatic-sufficient phenotype was consistent with her late diagnosis and relatively preserved pulmonary course.

Despite her advanced age, pulmonary function testing showed only mild obstructive physiology. Spirometry demonstrated FVC 1.95 L, 85% predicted; FEV1 1.33 L, 89% predicted; and FEV1/FVC ratio of 68%. The interpreting physician described stage I obstruction, with FEV1 stable compared with the prior year. Lung volume data showed RV/TLC of 45%, suggesting possible air trapping.

Taken together, upper and lower airway disease, characteristic airway microbiology, preserved pancreatic function, and an intermediate sweat chloride value form a coherent picture of partial rather than absent CFTR function. No single finding is diagnostic in isolation, and several, particularly the intermediate sweat chloride value and pancreatic sufficiency, would individually argue against CF. However, it is their convergence across multiple organ systems that is consistent with residual-function CF.

Eight months before her current presentation, she was started on vanzacaftor (20 mg), tezacaftor (100 mg), and deutivacaftor (250 mg) daily and reported symptomatic improvement after therapy. Airway clearance with an oscillatory positive expiratory pressure device was continued, and levalbuterol was used instead of albuterol because of prior severe tachycardia. Ongoing care included respiratory culture surveillance, nutritional and weight monitoring because of her low body mass index and risk of weight loss, otolaryngology follow-up for chronic sinus disease and nasal polyps, and endocrinology follow-up for osteoporosis with prior thoracic compression fractures treated with kyphoplasty.

Because the throat culture grew *Pseudomonas aeruginosa *(*P. aeruginosa*), longitudinal surveillance was indicated to determine whether this represented early acquisition, intermittent infection, or evolving chronic colonization. Given the low-burden throat culture and preserved lung function, the finding required clinical correlation with symptoms, imaging, spirometry trend, and subsequent microbiology.

## Discussion

This case illustrates the expanding clinical spectrum of CF and the importance of recognizing atypical CF phenotypes across the lifespan. Historically, CF was recognized as a severe childhood disorder characterized by pancreatic destruction, malnutrition, recurrent respiratory infections, and early death [3]. The development of sweat chloride testing, discovery of CFTR, expansion of newborn screening, advances in airway clearance and antimicrobial therapy, and emergence of CFTR modulators have substantially altered the disease course [1,2,7,10,11].

The patient's presentation is unusual because of her advanced age, late diagnosis, pancreatic sufficiency, intermediate sweat chloride value, and preserved lung function. Diagnosis at approximately age 62 suggests a milder phenotype that likely remained unrecognized for decades. This is consistent with prior observations that adults with residual CFTR function may lack classic childhood manifestations and may instead present with bronchiectasis, sinus disease, recurrent respiratory infections, NTM disease, or gastrointestinal symptoms [5,6]. Prior case reports have similarly emphasized that CF can be diagnosed in older adults and should not be excluded solely on the basis of age [12].

The intermediate sweat chloride value is central to this case. A value of 41 mmol/L is not diagnostic in isolation but falls within the intermediate range described in CF diagnostic guidelines. Farrell et al. [2] recommend that individuals with intermediate sweat chloride results undergo further evaluation with repeat sweat chloride testing, CFTR genetic analysis, and clinical correlation. In this patient, chronic bronchiectasis, chronic sinusitis with nasal polyps, prior NTM infection, and verified CFTR variants were consistent with a CF diagnosis despite the absence of pancreatic insufficiency.

Pancreatic sufficiency likely contributed to delayed recognition. Many patients with classic CF develop pancreatic insufficiency early in life; however, individuals with residual CFTR function may retain exocrine pancreatic function and present later [6]. This patient's normal fecal elastase and lack of response to pancreatic enzyme therapy supported pancreatic sufficiency.

This patient's presentation warrants comparison with the current definition of CFTR-related disorder (CFTR-RD). CFTR-RD describes a clinical entity associated with CFTR dysfunction that does not meet diagnostic criteria for CF, with recognized phenotypes characteristically confined to a single organ system [5,6]. Her intermediate sweat chloride value, pancreatic sufficiency, and identification of fewer than two confirmed CF-causing variants are all compatible with CFTR-RD. However, her disease involves multiple organ systems, comprising bronchiectasis, chronic rhinosinusitis with nasal polyps, and prior NTM infection; chronic rhinosinusitis with polyposis is not a recognized CFTR-RD phenotype. This multi-organ involvement, together with one unequivocally CF-causing variant, favors mild CF, although the distinction is recognized to be indistinct in individuals with residual CFTR function and CFTR-RD cannot be formally excluded on the available data.

The pulmonary findings also support a late-diagnosed CF phenotype. Spirometry demonstrated mild obstruction with FEV1 89% predicted and stability compared with the prior year, suggesting preserved lung function despite advanced age and chronic airway disease. CT imaging demonstrated middle-lung atelectasis/collapse with bronchiectasis, multifocal ground-glass and reticulonodular infiltrates, and small pleural effusions. While the ground-glass infiltrates and effusions were interpreted in the context of atypical/viral infection or multifocal pneumonia and possible congestive heart failure, the presence of bronchiectasis is consistent with chronic CF-related structural airway disease.

The new isolation of *P. aeruginosa* is clinically important. Chronic *P. aeruginosa* infection is associated with greater pulmonary disease burden in CF and often influences surveillance and treatment decisions. However, this patient's culture showed 1+ *P. aeruginosa* from a throat swab, and available records do not establish chronic colonization. Therefore, the finding should be interpreted cautiously as possible early acquisition, intermittent infection, or colonization requiring longitudinal follow-up. Repeat respiratory cultures, ideally from sputum if available, would help determine persistence and guide management.

Her prior history of MAC infection is also relevant. NTM infections are recognized in CF and may complicate bronchiectatic airway disease [5,6]. Although her concurrent acid-fast bacilli (AFB) stain was negative and preliminary culture showed no AFB isolated at less than one week, the specimen was a throat swab, and the laboratory report noted that swabs are suboptimal for mycobacterial culture. This limitation is important when interpreting negative AFB data.

This case also highlights the complexity of CF care in elderly patients. Standard CF care must be adapted to comorbidities, medication tolerance, frailty risk, and patient goals. Vertebral compression fractures, kyphosis, and kyphoplasty history limited tolerance of vest therapy, making handheld oscillatory positive expiratory pressure therapy more practical. Airway clearance remains a core component of CF management, but the optimal modality should be individualized based on tolerance, adherence, and clinical response [10]. Tachycardia with albuterol required use of levalbuterol. Low body mass index, recent weight loss, osteoporosis, cardiovascular disease, and chronic sinus disease all shaped management priorities.

Treatment with vanzacaftor/tezacaftor/deutivacaftor reflects the modern era of CF care. CFTR modulators have shifted treatment from management of downstream complications toward targeting the underlying CFTR protein defect. Earlier triple-combination therapy with elexacaftor/tezacaftor/ivacaftor demonstrated improvement in lung function and sweat chloride among eligible patients [7,8]. Vanzacaftor/tezacaftor/deutivacaftor was later approved as a once-daily next-in-class CFTR modulator for patients with responsive variants [9]. In this patient, reported symptomatic improvement after therapy supports the potential clinical relevance of CFTR modulator treatment even in selected older adults with late-diagnosed disease. In older adults, modulator therapy should still be individualized based on genotype eligibility, expected benefit, tolerability, hepatic monitoring, drug interactions, nutritional status, and comorbid conditions.

CFTR modulators target the defective protein itself rather than downstream disease manifestations. Correctors, tezacaftor/elexacaftor/vanzacaftor, improve folding and trafficking of misfolded CFTR, addressing the principal defect of F508del, in which the protein is degraded before reaching the cell surface. Potentiators, ivacaftor and deutivacaftor, increase the open probability of channels already at the membrane. Because F508del causes both trafficking and gating defects, combination therapy is required, and the addition of a second corrector produced the greater efficacy seen with triple-combination regimens [7,8]. Deutivacaftor is dosed once daily, reducing treatment burden compared with the twice-daily ivacaftor component of elexacaftor/tezacaftor/ivacaftor therapy [9].

Eligibility is determined by the patient's age, clinical diagnosis, and CFTR genotype. In the United States, elexacaftor/tezacaftor/ivacaftor is approved for patients aged two years and older, and vanzacaftor/tezacaftor/deutivacaftor for patients aged six years and older, when a patient has a clinical diagnosis of CF and at least one CFTR variant that is responsive based on clinical or in vitro data or results in production of CFTR protein [13,14]. Patients with two variants that produce no functional CFTR protein are unlikely to benefit from current potentiator-corrector combinations. For rare or incompletely characterized variants, consultation with a specialized CF center, review of current regulatory labeling and CFTR variant databases, and, where available, laboratory assessment of variant responsiveness may help guide eligibility.

In this patient, the presence of F508del provides a biologically plausible target for corrector-potentiator therapy, although the absent variant-phase information and incomplete classification of C76W limit genotype-phenotype interpretation. Her reported symptomatic improvement after initiation of vanzacaftor/tezacaftor/deutivacaftor suggests potential clinical benefit but should not be interpreted as objective proof of treatment response. Assessment would be strengthened by serial measurements of symptoms, spirometry, weight, exacerbation frequency, sweat chloride, treatment adherence, and adverse effects.

Despite their clinical benefits, CFTR modulators impose substantial financial burdens. Canada's Drug Agency estimated the annual cost of vanzacaftor/tezacaftor/deutivacaftor at CAD $322,151 per patient [15]. At these prices, sustained access depends almost entirely on insurance coverage, confidential negotiated pricing, or manufacturer patient-assistance programs. With such a high list price, any individuals, regardless of insurance status, could struggle to afford this medication. This is particularly relevant to older adults, in whom Medicare coverage rules, formulary placement, and prior-authorization requirements may differ from those applying to younger, privately insured patients. Financial strain persists despite insurance coverage. In a 2024 US survey of 936 people with CF and their care partners, 67% reported at least one financial issue caused by CF medical bills, including 47% of respondents with household incomes above 500% of the federal poverty level. More than one-quarter reported delaying or skipping medications (29%) or recommended care (25%) [16]. Insurance denials and copayment costs were each cited by 38% of those employing cost-saving strategies [16]. These tradeoffs occurred at all income levels and may carry long-term health consequences, a consideration relevant to any decision to initiate modulator therapy.

This case has several limitations. The phase and formal classification details of the reported CFTR variants were not available for inclusion in this manuscript. The original sweat chloride report and repeat testing history were also not available. Additionally, the available microbiology includes a throat culture rather than sputum culture, limiting interpretation of airway colonization. Longer follow-up after treatment with vanzacaftor/tezacaftor/deutivacaftor would strengthen assessment of clinical response, including symptoms, weight, exacerbation frequency, sweat chloride, and spirometric trajectory.

## Conclusions

We describe a woman diagnosed with CF at approximately 62 years of age and followed to age 87, whose course was characterized by pancreatic sufficiency, an intermediate sweat chloride value of 41 mmol/L, chronic bronchiectasis, chronic sinusitis with nasal polyps, prior MAC infection, and mild obstructive physiology that remained stable relative to the prior year.

Several features of this case limit the strength of the inferences that can be drawn. The diagnosis was established at an outside institution, and neither the original sweat chloride documentation nor repeat testing was available for review. CFTR analysis identified F508del together with a rare C76W variant, but variant phase and formal classification could not be obtained; on the data available, mild CF cannot be formally distinguished from a CFTR-related disorder phenotype. A single low-burden throat culture grew *P. aeruginosa*, which requires longitudinal surveillance rather than representing established chronic infection. The patient reported symptomatic improvement eight months after initiation of vanzacaftor/tezacaftor/deutivacaftor, although this response was subjective and was not accompanied by documented change in spirometry, sweat chloride, weight, or exacerbation frequency.

As a single case, these observations are hypothesis-generating and cannot establish prevalence, prognosis, or treatment effect. They nonetheless illustrate that CF may be recognized in later adulthood in patients with preserved pancreatic function and near-normal spirometry, and they support consideration of CFTR testing in adults with otherwise unexplained bronchiectasis, chronic sinus disease with nasal polyps, or nontuberculous mycobacterial infection.
